# Cardiac Troponin I Reveals Diagnostic and Prognostic Superiority to Aminoterminal Pro-B-Type Natriuretic Peptide in Sepsis and Septic Shock

**DOI:** 10.3390/jcm11216592

**Published:** 2022-11-07

**Authors:** Jan Forner, Tobias Schupp, Kathrin Weidner, Jonas Rusnak, Schanas Jawhar, Floriana Dulatahu, Lea Marie Brück, Michael Behnes, Ursula Hoffmann, Thomas Bertsch, Maximilian Kittel, Ibrahim Akin

**Affiliations:** 1 First Department of Medicine, University Medical Centre Mannheim, Faculty of Medicine Mannheim, Heidelberg University, 61867 Mannheim, Germany; 2 European Center for AngioScience (ECAS) and German Center for Cardiovascular Research (DZHK) Partner Site Heidelberg/Mannheim, 68167 Mannheim, Germany; 3 Institute of Clinical Chemistry, Laboratory Medicine and Transfusion Medicine, Nuremberg General Hospital, Paracelsus Medical University, 90419 Nuremberg, Germany; 4 Institute for Clinical Chemistry, Faculty of Medicine Mannheim, Heidelberg University, 68167 Mannheim, Germany

**Keywords:** sepsis, septic shock, NT-pro BNP, cTNI, prognosis

## Abstract

Data regarding the prognostic value of cardiac biomarkers in patients suffering from sepsis or septic shock is scarce. Studies investigating the prognostic role of cardiac biomarkers in patients with sepsis and septic shock were commonly published prior to the sepsis-3 criteria and were often not restricted to septic patients only, too. This study investigated the diagnostic and prognostic value of the aminoterminal pro-B-type Natriuretic Peptide (NT-pro BNP) and cardiac troponin I (cTNI) in patients with sepsis and septic shock. Consecutive patients with sepsis and septic shock were included from 2019 to 2021. Blood samples were retrieved from the day of disease onset (i.e., day 1), day 2 and 3. Firstly, the diagnostic value of the NT-pro BNP and cTNI to diagnose sepsis or septic shock was tested. Secondly, the prognostic value of the NT-pro BNP and cTNI was examined with regard to the 30-day all-cause mortality. The statistical analyses included univariable *t*-tests, Spearman’s correlations, C-statistics, Kaplan–Meier analyses and Cox proportional regression analyses. A total of 162 patients were included prospectively, of which 57% had a sepsis and 43% a septic shock. The overall rate of all-cause mortality at 30 days was 53%. With an area under the curve (AUC) of 0.658 on day 1 and 0.885 on day 3, cTNI expressed a better diagnostic value than NT-pro BNP, especially on day 3 (ΔAUCd3 = 0.404; *p* = 0.022). Furthermore, cTNI displayed a moderate but slightly better prognostic value than NT-pro BNP on all examined days (AUC for cTNI, d1 = 0.635; 95% CI 0.541–0.729; *p* = 0.007 vs. AUC for NT-pro BNP, d1 = 0.582; 95% CI 0.477–0.687; *p* = 0.132). In conclusion, cTNI was a reliable diagnostic parameter for the diagnosis of sepsis and septic shock, as well as a reliable prognostic tool with regard to 30-day all-cause mortality in patients suffering from sepsis and septic shock.

## 1. Introduction

Sepsis is defined as a life-threatening organ dysfunction caused by a dysregulated host response as a consequence of an infection [1]. Since the publication of the so-called “sepsis-3” criteria in 2016, sepsis-related organ dysfunction is diagnosed by using the “Sequential Organ Failure Assessment” (SOFA) score [1,2]. Within the SOFA score, the cardiovascular system is only assessed by using mean arterial pressure (MAP) and the need for catecholamine infusions, whereas the use of cardiac biomarkers is not included in the decision-making of sepsis. However, myocardial dysfunction was shown to be common in septic patients, and patients with septic shock frequently show reversible left ventricular dysfunction [3,4].

Natriuretic peptides are released as a consequence of increased myocardial wall stress and volume status. The secretion of natriuretic peptides, such as the B-type natriuretic peptide (BNP), leads to relaxation of the vasomotor tone and inhibition of sympathetic activity, ultimately resulting in increases in natriuresis and diuresis [5,6]. The aminoterminal fragment (NT-pro-BNP) is biologically inert; however, both BNP and NT-pro-BNP are considered important biomarkers for the diagnosis-making and risk stratification in patients with heart failure (HF) [7,8]. The cardiac troponin complex—consisting of troponin C (cTNC), troponin T (cTNT) and troponin I (cTNI)—is involved in the regulation of cardiac muscle contraction. The regulatory role of cTNI consists of the inhibition of the adenosine 5′-triphosphatase (ATPase) activity of the actomyosin complex and the modulation of cross-bridge formation and cardiac muscle contraction [9,10]. In particular, cTNT and cTNI are biomarkers of myocardial injury and are commonly released during myocardial necrosis in the presence of an acute myocardial infarction (AMI) [11], which further promotes adverse cardiac remodeling [12]. Both NT-pro BNP and cTNI were shown to be increased in patients with non-cardiac diseases, including pulmonary hypertension, chronic obstructive pulmonary disease (COPD), renal dysfunction and sepsis [13].

Studies investigating the prognostic role of NT-pro BNP and cTNI were commonly published prior to the “sepsis-3” criteria and predominantly limited to a small study population [14,15,16]. Hence, the present study aimed to investigate the diagnostic and prognostic value of NT-pro BNP and cTNI in patients suffering from sepsis and septic shock.

## 2. Materials and Methods

### 2.1. Study Patients, Design and Data Collection

The present study prospectively included all consecutive patients presenting with sepsis or septic shock on admission to the internal ICU at the University Medical Center Mannheim, Germany, from June 2019 to January 2021. All relevant clinical data related to the index event was documented by using the electronic hospital information system, as well as the IntelliSpace Critical Care and anesthesia information system (ICCA, Philips, Philips GmbH Market DACH, Hamburg, Germany) implemented at the ICU, organizing patient data such as admission documents, vital signs, laboratory values, treatment data and consult notes.

The presence of sepsis and septic shock, as well as important laboratory data, sepsis-related scores, hemodynamic measurements, and ventilation parameters were assessed upon disease onset (i.e., day 1), day 2 and day 3.

Moreover, baseline characteristics, prior medical history, length of index hospital stay, pharmacological therapies and data derived from imaging diagnostics were documented. The documentation of source data was performed by intensivists and ICU nurses during routine clinical care.

The present study was derived from an analysis of the “Mannheim Registry for Sepsis and Septic Shock” (MARSS-registry) [17], which represents a prospective single-center registry including all consecutive patients presenting with sepsis or septic shock at the ICU for internal medicine of the University Medical Center Mannheim (UMM), Germany (clinicaltrials.gov identifier: NCT05231720). The registry was established according to the principles of the declaration of Helsinki and was approved by the medical ethics committee II of the Medical Faculty Mannheim, University of Heidelberg, Germany.

### 2.2. Inclusion and Exclusion Criteria, Study Endpoints

For the present study, all consecutive patients with sepsis and septic shock were included. Patients without evidence of the NT-pro BNP and the cTNI on sepsis day 1 were excluded from the present study. Furthermore, patients without measurement of the left ventricular ejection fraction (LVEF) were excluded.

The diagnosis of sepsis and septic shock was determined according to the “Third International Consensus Definition for Sepsis and Septic Shock” (i.e., sepsis-3) [1]. Accordingly, sepsis was defined as a life-threatening organ dysfunction, caused by a dysregulated host response to an infection. Organ dysfunction was defined as an increase of ≥2 in the Sequential Organ Failure Assessment (SOFA) score. Septic shock was defined as persistent hypotension, despite adequate volume resuscitation, requiring vasopressors to maintain a mean arterial pressure (MAP) ≥ 65 mm Hg and a serum lactate ≥ 2 mmol/L [1]. Values of LVEF were retrieved from standardized transthoracic echocardiographic examinations commonly performed during ICU hospitalization to assess realistic LVEF values beyond the acute phase of septic cardiomyopathy. LVEF measurements were performed in two- and four-chamber apical projections during routine clinical care and calculated by using the Simpson’s biplane method, according to the European guidelines [18].

All-cause mortality at 30 days was documented by using our electronic hospital information system and by directly contacting state resident registration offices (‘bureau of mortality statistics’). The identification of patients was verified by place of name, surname, day of birth, and registered living address. No patient was lost to follow-up with regard to all-cause mortality at 30 days.

### 2.3. Measurement of NT-Pro BNP and cTNI

First, cTNI was measured with the SIEMENS Atellica Solution CH 930™. The lowest detection limit of the assay was 0.015 ng/mL, with a linearity range from 0.025 to 25 ng/mL. The 99th percentile, which was measured from a healthy reference population, was 0.045 ng/mL, with a coefficient of variation of 10% [19,20]. NT-pro BNP determinations were performed as a direct chemiluminescence sandwich immunoassay on the Atellica Solution IM (Siemens Healthineers, Erlangen, Germany). The linear quantification range of the assay for serum and plasma is 35–35,000 pg/mL (4.13–4130 pmol/L). The clinical decision threshold for the NT-pro BNP assay to separate healthy from sick patients is 125 pg/mL for patients aged <75 years and 450 pg/mL for patients aged ≥75 years.

### 2.4. Statistical Methods

Quantitative data is presented as the median and interquartile range (IQR). Comparisons were applied by using Student’s *t*-test for normally distributed data or the Mann–Whitney U test for nonparametric data. Deviations from a Gaussian distribution were tested by using the Kolmogorov–Smirnov test. Qualitative data are presented as absolute and relative frequencies and were compared by using the Chi-square test. Spearman’s rank correlation for nonparametric data was used to examine the association of the NT-pro BNP and the cTNI with medical and laboratory parameters measured on day 1.

#### 2.4.1. Diagnostic Performance of NT-Pro BNP and cTNI

Within the entire study cohort, C-statistics were applied with the calculation of the receiver operating characteristics (ROCs) and the corresponding area under the curves (AUCs) to assess the ability of NT-pro BNP and cTNI to discriminate between patients with sepsis and septic shock (diagnostic performance) on days 1, 2 and 3. AUCs for diagnostic performance were compared by the method of Hanley et al. [21].

#### 2.4.2. Prognostic Performance of NT-Pro BNP and cTNI

Within the entire study cohort, C-statistics were applied on days 1, 2 and 3, with calculation of ROCs and the corresponding AUCs for 30-day all-cause mortality (prognostic performance). AUCs for prognostic performance were compared by the method of Hanley et al. [21]. Kaplan–Meier analyses according to NT-pro BNP and cTNI were performed within the entire study cohort, as well as stratified for LVEF. Univariable hazard ratios (HRs) were calculated together with their respective 95% confidence intervals. Thereafter, multivariable Cox regression models were developed by using the “forward selection” option, where only statistically significant variables (*p* < 0.05) were included and analyzed simultaneously.

Results of all statistical tests were considered significant for *p* ≤ 0.05. SPSS (Version 25, IBM, Armonk, NY, USA) and GraphPad Prism (Version 9, GraphPad Software, San Diego, CA, USA) were used for statistics.

## 3. Results

### 3.1. Study Population

**Figure 1:**
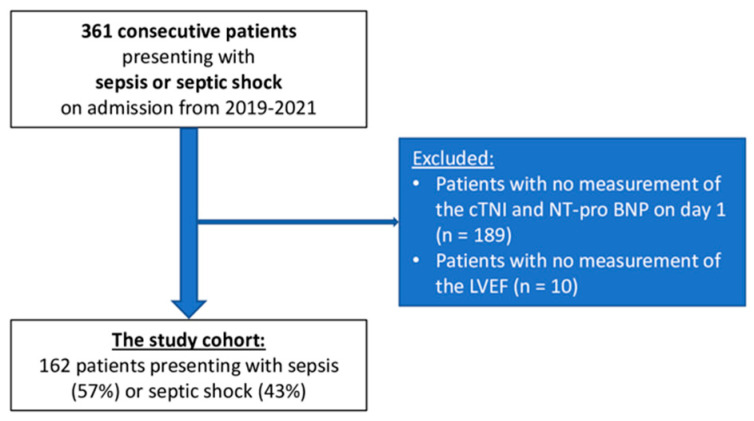
Study population.

From a total of 361 consecutive patients with sepsis or septic shock, 189 patients with no measurement of the NT-pro BNP and cTNI on day 1 were excluded. Additionally, 10 patients with no evidence of the LVEF were excluded. The final study cohort comprised 162 patients, of which 57% presented with sepsis and 43% with septic shock on day 1 (Figure 1, flowchart).

The median NT-pro BNP level on day 1 was 2794 pg/mL (IQR 913–7978 pg/mL), and the median cTNI level was 0.14 µg/mL (IQR 0.03–0.92 µg/mL). As depicted in Table 1, the median age was 70 years, and most patients were males (65%). When stratified for patients presenting with sepsis or septic shock (Table 1, middle and right panel), the rates of coronary artery disease (39% vs. 45%; *p* = 0.427), congestive heart failure (19% vs. 30%; *p* = 0.103) and atrial fibrillation (27% vs. 36%; *p* = 0.203) were equally distributed. Patients with sepsis presented more often with LVEF 45–54% (38% vs. 13%; *p* = 0.001), whereas a LVEF 35–44% was more common in patients with septic shock (16% vs. 29%; *p* = 0.049). Patients with LVEF ≥ 55% (28% vs. 29%; *p* = 0.886) and LVEF < 35% (18% vs. 29%) were equally common in both groups. Moreover, patients with septic shock presented with higher rates of cardiopulmonary resuscitation (5% vs. 30%; *p* = 0.001).

As illustrated in Table 2, a pulmonary infectious focus was the most common source of infection in both groups (57% vs. 58%), followed by an unknown focus of infection (20% vs. 26%). With regard to sepsis-related scores, the “Disseminated Intravascular Coagulation” (DIC) score (median 1 vs. 2; *p* = 0.001), the “acute physiology and chronic health evaluation II” (APACHE II) score (median 20 vs. 27; *p* = 0.001), the acute physiology score (median 13 vs. 22; *p* = 0.001) and the SOFA score (median 9 vs. 13; *p* = 0.001) were higher in patients with septic shock. Furthermore, patients presenting with septic shock required the use of catecholamines (79% vs. 99%; *p* = 0.001) and mechanical ventilation (52% vs. 68%; *p* = 0.048) more frequently than septic patients. Patients with septic shock revealed increased serum lactate levels (median 1.4 mmol/L vs. 3.5 mmol/L; *p* = 0.001) and higher international normalized ratios (INR) (median 1.1 vs. 1.3; *p* = 0.001). The cTNI levels (median 0.08 vs. 0.37 µg/L; *p* = 0.002) were significantly higher in patients admitted with septic shock, whereas the NT-pro BNP levels did not significantly differ between both groups (median 2256 vs. 4500 pg/mL; *p* = 0.085). Finally, all-cause mortality at 30 days occurred more often in patients with septic shock on admission (42% vs. 59%; *p* = 0.028) (Table 2).

### 3.2. Association of cTNI and NT-Pro BNP with Clinical and Laboratory Data

Table 3 illustrates the correlation of cTNI and NT-pro BNP on day 1 with clinical and laboratory data. The cTNI showed a significant, yet negligible, correlation with procalcitonin (r = 0.209; *p* = 0.029), as well as sepsis-related scores, such as the SOFA score (r = 0.201; *p* = 0.020), acute physiology score (r = 0.239; *p* = 0.006) and APACHE II score (r = 0.220; *p* = 0.011). Furthermore, cTNI displayed a low positive correlation with the LVEF (r = 0.307; *p* = 0.001). The correlation between cTNI and NT-pro BNP was moderately positive (r = 0.528; *p* = 0.001). The NT-pro BNP presented a negligible correlation with age (r = 0.222; *p* = 0.018), platelet count (r = −0.225; *p* = 0.016), procalcitonin (r = 0.247; *p* = 0.012), PaO_2_/FiO_2_ ratio (r = 0.202; *p* = 0.037), creatinine (r = 0.291; *p* = 0.002) and MAP (r = −0.199; *p* = 0.034). The correlation between NT-pro BNP and sepsis-related scores, such as the SOFA score (r = 0.226; *p* = 0.015), acute physiology score (r = 0.192; *p* = 0.040) and APACHE II score (r = 0.281; *p* = 0.002), was significant but negligible as well. Moreover, the NT-pro BNP depicted a low negative correlation with mechanical ventilation days (r = −0.342; *p* = 0.001) and intensive-care days (r = −0.304; *p* = 0.001), while the correlation with the LVEF was low positive (r = 0.439; *p* = 0.001).

### 3.3. Diagnostic Performance of cTNI and NT-Pro BNP

**Figure 2:**
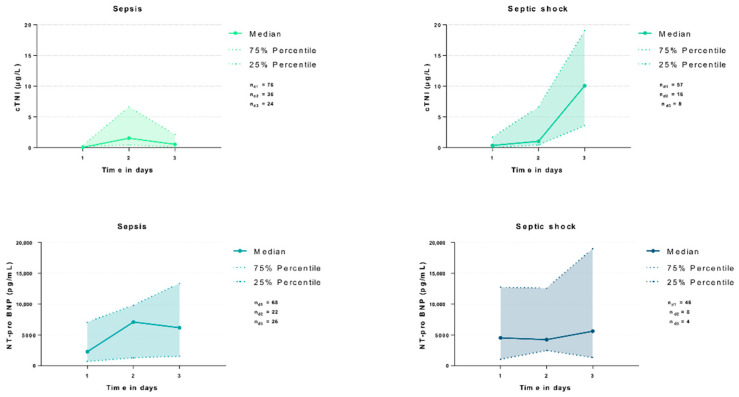
Distribution of cTNI and NT-pro BNP among patients with sepsis and septic shock during the first 3 days of sepsis.

The distributions of the cTNI and NT-pro BNP during the initial 3 days of sepsis and septic shock are presented in Figure 2. The cTNI levels were significantly higher in patients with septic shock as compared to septic patients on day 1 (median 0.08 vs. 0.37 µg/L; *p* = 0.002) and day 3 (median 0.56 vs. 10.10 µg/L; *p* = 0.001), whereas the NT-pro BNP levels did not differ significantly between both groups on all evaluated ICU treatment days (*p* > 0.05).

With an AUC of 0.658 on day 1, the cTNI was able to discriminate between patients with sepsis and septic shock, whereas the NT-pro BNP displayed a poor diagnostic value (AUC 0.595). Especially on day 3, the cTNI revealed a good diagnostic value, which was superior to the diagnostic value of the NT-pro BNP (AUC 0.885 vs. 0.481; ΔAUC = 0.404; *p*-value for the AUC difference = 0.022) (Table 4).

### 3.4. Prognostic Performance of cTNI and NT-Pro BNP

**Figure 3:**
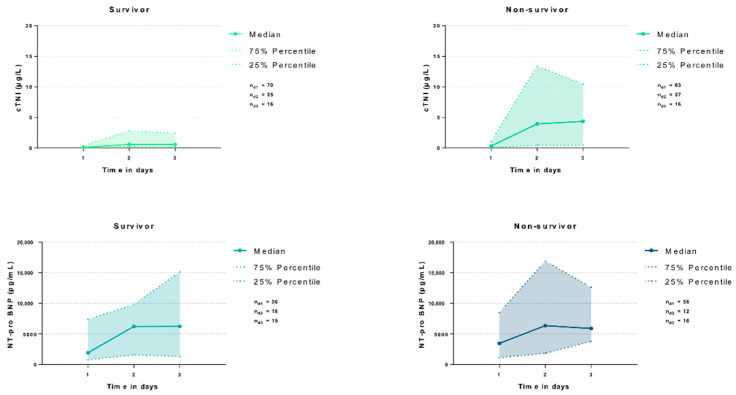
Distribution of cTNI and NT-pro BNP among patients on days 1, 2 and 3, comparing 30-day survivors and non-survivors.

Overall, the risk of 30-day all-cause mortality was 49%. During the first 3 days of ICU hospitalization, cTNI levels were higher among 30-day non-survivors as compared to survivors on day 1 (median 0.09 vs. 0.31 µg/L; *p* = 0.007) and day 2 (median 0.59 vs. 3.93 µg/L; *p* = 0.021), but not on day 3 (median 0.57 vs. 4.34 µg/L; *p* = 0.200). In contrast, NT-pro BNP levels did not significantly differ between non-survivors and survivors on all evaluated ICU treatment days (*p* > 0.05) (Figure 3).

The prognostic AUCs of cTNI were statistically significant during the first two days of ICU treatment (AUC 0.635–0.687). Of note, prognostic AUCs for NT-pro BNP were not statistically significant on the evaluated treatment days (Table 5).

**Figure 4:**
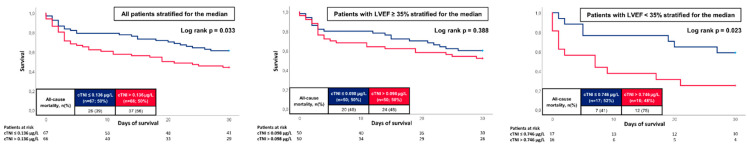
Kaplan–Meier curves for cTNI according to all-cause mortality at 30 days within the entire study cohort (**left**), in patients with LVEF ≥ 35% (**middle**) and LVEF < 35% (**right**).

**Figure 5:**
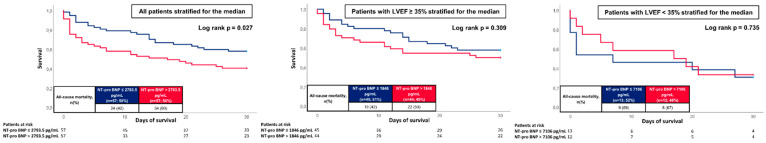
Kaplan–Meier curves for NT-pro BNP according to all-cause mortality at 30 days within the entire study cohort (**left**), in patients with LVEF ≥ 35% (**middle**) and in patients with LVEF < 35% (**right**).

At 30 days, all-cause mortality occurred in 56% of the patients with cTNI levels above the median and in 39% of the patients with cTNI levels below or equal to the median. Accordingly, the risk of all-cause mortality was increased in patients with elevated cTNI levels (log rank *p* = 0.033; HR = 1.703; 95% CI 1.030–2.814; *p* = 0.038) (Figure 4, left panel). Specifically, an increased all-cause mortality was observed in patients with higher cTNI levels presenting with a LVEF < 35% (41% vs. 75%, log rank *p* = 0.023; HR = 2.778; 95% CI 1.084–7.122; *p* = 0.033), but not in patients with a LVEF ≥ 35% (40% vs. 48%, log rank *p* = 0.388; HR = 1.293; 95% CI 0.714–2.341; *p* = 0.397) (Figure 4, middle and right panel). In line, NT-pro BNP levels above the median were associated with an increased risk of 30-day all-cause mortality (42% vs. 60%, log rank *p* = 0.027; HR = 1.769; 95% CI 1.048–2.986; *p* = 0.033). However, this was no longer observed after stratification for LVEF < 35% (69% vs. 67%, log rank *p* = 0.735; HR = 0.853; 95% CI 0.328–2.217; *p* = 0.745) and LVEF ≥ 35% (42% vs. 50%, log rank *p* = 0.309; HR = 1.367; 95% CI 0.739–2.526; *p* = 0.319) (Figure 5).

### 3.5. Multivariable Cox Regression Models

After multivariable adjustment, high cTNI levels (HR = 2.251; CI 1.017–4.981; *p* = 0.045) and a severely impaired LVEF (HR = 4.048; CI 1.475–11.304; *p* = 0.007) were associated with an increased risk of 30-day all-cause mortality, while high NT-pro BNP levels (HR = 1.364; CI 0.528–3.521 *p* = 0.522) were not (Table 6). Furthermore, concomitant malignancies (HR = 3.439; *p* = 0.008) displayed an increased risk of 30-day all-cause mortality.

### 3.6. NT-Pro BNP Adjusted for eGFR

Finally, the diagnostic and prognostic impacts of the eGFR adjusted NT-pro BNP levels were re-evaluated. It was demonstrated that the diagnostic (AUC 0.517 vs. 0.595) and prognostic (AUC 0.579 vs. 0.582) values did not differ when comparing the adjusted to the unadjusted NT-pro BNP levels (not demonstrated).

## 4. Discussion

The present study investigated the diagnostic and prognostic value of NT-pro BNP and cTNI in a well-defined and selective group of internistic patients presenting with sepsis and septic shock. This study suggests that the cTNI is a reliable diagnostic parameter to distinguish between patients with sepsis or septic shock. Moreover, it indicates that the diagnostic performance of the cTNI is superior to the NT-pro BNP. Furthermore, cTNI displayed a moderate prognostic value with regard to 30-day all-cause mortality. Both cTNI and NT-pro BNP levels above their respective median were associated with an increased risk of 30-day all-cause mortality. The prognostic impact of high cTNI levels was still evident in patients admitted with LVEF < 35%.

The exact underlying pathophysiology of elevated cardiac biomarkers (such as NT-pro BNP and cTNI) in patients with sepsis or septic shock remains unclear. In the past, various molecules, for example interleukin-6 (IL-6) or tumor necrosis factor-a, have been proposed as agents contributing to septic cardiomyopathy [22,23]. Furthermore, cardiac biomarkers may be increased as a result of increased wall stress, myocardial oxygen demand–supply mismatch (i.e., type 2 myocardial infarction), drug toxicity or renal failure [24]. It has also been suggested, that myocardial depressant molecules increase the permeability of myocytes, thereby leaking cTNI into the bloodstream [14]. Ultimately, the pathomechanism appears to be different to thrombus-associated myocardial damage, as implied by a study of Altmann et al. from 2010, finding no differences in coagulation parameters analyzed with rotational thromboelastometry in patients with systemic inflammatory response syndrome (SIRS), sepsis and septic shock presenting with and without elevated cTNI levels [25]. Thus, in septic patients, troponin elevation may occur in the absence of myocardial ischemia. This is confirmed by studies suggesting reversible myocardial dysfunction in sepsis survivors, as well as three studies that found flow-limiting CAD (assessed by stress echocardiography, coronary angiography or postmortem examination) in only 4% to 6% of patients with sepsis or septic shock [14,25,26]. However, no guidelines for the treatment of patients with elevated cTNI levels are available [27]. 

Although the prognostic impact of cTNT and cTNI was investigated within various clinical studies, suggesting increased risk of in-hospital and ICU mortality in patients with elevated troponin levels, most studies are limited to a retrospective study design and a non-consecutive inclusion of patients [28,29]. For instance, in a retrospective observational cohort study, Wen et al. demonstrated a reliable prognostic performance of the cTNT for the prediction of 28-day mortality with a sensitivity of 75.0% and a specificity of 61.7% for the optimal cutoff value (i.e., 0.039 ng/mL) in patients with sepsis and septic shock admitted to an ICU. Further on, cTNT, in combination with the red-cell-distribution width, was able to improve the prognostic performance of the SOFA score in this study [30]. Those results are contrasted by the randomized “Vasopressin and Septic Shock Trial” (VASST), which included 121 patients treated with vasopressin or norepinephrine, and for which no association of cTNI or cTNT with the risk of 28-day mortality was found [15]. NT-pro BNP was demonstrated to be associated with an increased risk of short-term all-cause mortality within multiple clinical studies. For instance, in a prospective multicenter study, also including patients treated at our clinic between 2001 and 2002, Brückmann et al. suggested that septic patients with NT-pro BNP levels > 1400 pmol/L were at a 3.9-fold increased risk of death as compared to patients with lower NT-pro BNP levels [31]. In line with this, BNP was found to be superior to the SOFA score in predicting 90-day all-cause mortality in 259 septic patients with no evidence of concomitant heart failure [32]. The prognostic value of both BNP and cTNI was comprehensively investigated in a study comprising 233 patients with sepsis and concomitant cancer. The BNP was an independent predictor of 28-day all-cause mortality, but cTNI was not. Interestingly, BNP on sepsis day 3 (cutoff 681.5 pg/mL) revealed a sensitivity of 91.5% and a specificity of 88.7% [33]. Furthermore, the dynamic changes of NT-pro BNP during the first week after trauma were investigated within a prospective study including 60 major trauma patients. The study demonstrated that NT-pro BNP levels were higher in non-survivors than in survivors and increased during the week in non-survivors [34]. A study by Andersson et al. from 2019 examined the high-sensitivity cTNT (hs-cTNT) as an independent predictor of 30-day mortality in 856 patients with cardiac arrest, sepsis, heart failure and respiratory failure. They found the hs-cTNT to display a reliable prognostic value in septic patients, while hs-cTNT showed poor prognostic ability in patients with cardiac arrest, heart failure and respiratory failure [35]. Those findings are in line with a study by de Groot et al., revealing hs-cTNT as an independent predictor of in-hospital mortality in 292 patients. However, this study was not restricted to septic patients [36]. The present study, consecutively including patients treated at an internistic ICU with sepsis or septic shock according to the sepsis-3 criteria, suggests a poor prognostic accuracy of NT-pro BNP, whereas cTNI revealed a moderate predictive value with regard to 30-day all-cause mortality.

Only a few data are available that focus on the prognostic value of cardiac biomarkers while also controlling for cardiac function. The association of cTNI and left ventricular (LV) dysfunction was investigated in 46 patients with septic shock undergoing transesophageal echocardiography, and a strong association between LV dysfunction and cTNI positivity was demonstrated [16]. In line with this, Mehta et al. found lower LVEF, higher incidence of regional wall motion abnormalities and higher mortality in patients with elevated cTNI levels in 37 patients presenting with septic shock [37]. Moreover, a study by Hai et al. from 2021 found an association between high levels of hs-cTNT and left ventricular systolic dysfunction (LVSD), as measured by speckle-tracking echocardiography, in 116 patients with sepsis or septic shock [38]. Those results are also in line with a monocentric prospective study from 2022, finding high-sensitivity cTNI (hs-cTNI) and NT-pro BNP to be related to a risk for LVSD in 124 septic patients [39]. Correspondingly, the prognostic impact of myocardial dysfunction, as assessed by fractional area contraction (FAC), was examined within a small study including 34 patients with severe sepsis or septic shock. Increased BNP levels on day 2 were observed in patients with FAC < 50% and were associated with increased risk of ICU mortality [3]. Within the present study, both cTNI and NT-pro BNP correlated with the LVEF in patients with sepsis or septic shock. However, when stratified for LVEF < 35%, only high cTNI levels were associated with 30-day all-cause mortality, whereas high NT-pro BNP levels were not associated with the prognostic outcome.

This study has several limitations. Due to the single-center observational study design, results may be influenced by measured and unmeasured confounding factors, although we adjusted for potential confounders using multivariable Cox regression. On day 2 and 3 of follow-up, cTNI and NT-pro BNP levels were not available for every patient, which may have accounted for selection bias. Furthermore, follow-up cTNI and NT-pro BNP levels on ICU hospitalization day 5 to 10 were only available in a minor part of the study population and, therefore, were not taken into account within the present study. With regard to the diagnostic value of NT-pro BNP and cTNI, no control group with healthy individuals was considered. Data on the extent of concomitant CAD, as well as PCI-related data, were not assessed for the present study. Finally, the effects of NT-pro BNP and cTNI on long-term outcomes were beyond the scope of the present study.

In conclusion, the present study identifies cTNI superior compared to NT-pro BNP with regard to the diagnosis of septic shock. In line, both increasing cTNI and NT-pro BNP levels were associated with increased risk of 30-day all-cause mortality. The prognostic impact of high cTNI levels was still evident in patients admitted with LVEF < 35%.

## Figures and Tables

**Table 1:** Baseline characteristics.

	All Patients (*n* = 162)		Sepsis (*n* = 93)		Septic Shock (*n* = 69)		*p*-Value
**Age, median; (IQR)**	70	(61–78)	70	(60–78)	70	(60–80)	0.966
**Male sex**, *n* (%)	106	(65.4)	61	(65.6)	45	(65.2)	0.961
**Body mass index** (kg/m^2^), median; (IQR)	26.67	(24.22–30.86)	26.58	(23.77–29.89)	26.73	(24.69–32.65)	0.410
**Entry criteria**, median; (IQR)							
Body temperature (°C)	36.8	(36–37.6)	36.9	(36.1–37.4)	36.6	(35.6–37.9)	0.381
Heart rate (bpm)	102	(87–115)	96	(85–111)	108	(90–123)	**0.027**
Systolic blood pressure (mmHg)	111	(96–129)	114	(99–133)	108	(88–125)	**0.010**
Respiratory rate (breaths/minute)	22	(18–26)	22	(18–26)	21	(18–26)	0.563
**Cardiovascular risk factors**, *n* (%)							
Arterial hypertension	113	(69.8)	65	(69.9)	48	(69.6)	0.964
Diabetes mellitus	60	(37.0)	35	(37.6)	25	(36.2)	0.855
Hyperlipidemia	51	(31.5)	24	(25.8)	27	(39.1)	0.071
Smoking	44	(27.3)	27	(29.3)	17	(24.6)	0.507
**Prior medical history**, *n* (%)							
Coronary artery disease	67	(41.4)	36	(38.7)	31	(44.9)	0.427
Congestive heart failure	39	(24.1)	18	(19.4)	21	(30.4)	0.103
Atrial fibrillation	50	(30.9)	25	(26.9)	25	(36.2)	0.203
Chronic kidney disease	32	(19.8)	22	(23.7)	10	(14.5)	0.147
COPD	32	(19.8)	18	(19.4)	14	(20.3)	0.882
Liver cirrhosis	7	(4.3)	4	(4.3)	3	(4.3)	0.988
Malignancy	48	(29.6)	26	(28.0)	22	(31.9)	0.588
Immunosuppression	19	(12.1)	13	(14.8)	6	(8.7)	0.247
**LVEF at admission, *n* (%)**							
≥55%	46	(28.4)	26	(28.0)	20	(29.0)	**0.886**
54–45	44	(27.2)	35	(37.6)	9	(13.0)	**0.001**
44–35%	35	(21.6)	15	(16.1)	20	(29.0)	**0.049**
<35%	37	(22.8)	17	(18.3)	20	(29.0)	**0.108**
**Cardiopulmonary resuscitation**, *n* (%)	26	(16.0)	5	(5.4)	21	(30.4)	**0.001**
In-hospital	7	(4.3)	2	(2.2)	5	(7.2)	**0.001**
Out-of-hospital	19	(11.7)	3	(3.2)	16	(23.2)	

COPD, chronic obstructive pulmonary disease; IQR, interquartile range; LVEF, left ventricular ejection fraction. Level of significance between sepsis and septic shock *p* < 0.05. Bold type indicates statistical significance.

**Table 2:** Sepsis-related data, follow-up data and endpoints.

	All Patients (*n* = 162)		Sepsis (*n* = 93)		Septic Shock (*n* = 69)		*p*-Value
**Sepsis scores, median; (IQR)**							
DIC	1	(1–2)	1	(0–2)	2	(1–3)	**0.001**
Acute physiology score	17	(12–23)	13	(8–19)	22	(15–25)	**0.001**
APACHE II	24	(18–30)	20	(14–27)	27	(21–33)	**0.001**
SOFA	10	(8–13)	9	(6–12)	13	(10–15)	**0.001**
ISARIC-4C-Mortality score	15	(12–16)	14	(12–16)	15	(12–16)	0.615
**Infection focus**, *n* (%)							
Pulmonary	93	(57.4)	53	(57.0)	40	(58.0)	0.216
Urogenital	19	(11.7)	15	(16.1)	4	(5.8)	
Intra-abdominal	12	(7.4)	6	(6.5)	6	(8.7)	
Wound	1	(0.6)	0	(0.0)	1	(1.4)	
Unknown	37	(22.8)	19	(20.4)	18	(26.1)	
SARS-CoV-2 infection, *n* (%)	24	(14.8)	19	(20.4)	5	(7.2)	**0.020**
**Multiple organ support during ICU**							
Vasopressor support norepinephrine, *n* (%)	141	(87.0)	73	(78.5)	68	(98.6)	**0.001**
Dose of norepinephrine (µg; median (IQR))	51.7	(5.8–158.3)	25.0	(1.8–104.3)	105.4	(24.3–281.5)	**0.001**
Dialysis during hospitalization, *n* (%)	75	(46.3)	31	(33.2)	44	(63.8)	**0.001**
Extracorporeal membrane oxygenation, *n* (%)	14	(8.6)	9	(9.7)	5	(7.2)	0.586
**Respiratory status**							
Mechanical ventilation, *n* (%)	96	(59.3)	49	(52.3)	47	(68.1)	**0.048**
Invasive mechanical ventilation, *n* (%)	73	(45.1)	29	(31.2)	44	(63.8)	**0.001**
Duration of mechanical ventilation (days; mean, (range))	5	(1–15)	5	(1–16)	3	(1–15)	0.715
PaO_2_/FiO_2_ ratio (median; (IQR))	191	(129–285)	192	(129–297)	191	(127–278)	0.866
PaO_2_ (median; (IQR))	91	(72–123)	87	(69–117)	97	(80–126)	0.081
**Liver function**							
Acute liver failure, *n* (%)	15	(9.3)	6	(6.5)	9	(13.0)	0.152
**Renal function**, median; (IQR)							
Serum creatinine (mg/dL)	1.9	(1.28–3.03)	1.69	(1.09–2.85)	2.16	(1.56–3.49)	**0.010**
GFR (mL/min)	31.49	(19.2–51.88)	34.93	(21.7–62.01)	26.87	(16.2–40.48)	**0.008**
Urine output (mL)	790	(179–1493)	900	(415–1650)	510	(40–1270)	**0.022**
Dialysis (days)	0	(0–4)	0	(0–4)	2	(0–6)	**0.001**
**Baseline laboratory values**, median; (IQR)							
pH	7.37	(7.28–7.42)	7.39	(7.31–7.44)	7.33	(7.22–7.40)	**0.001**
Lactate (mmol/L)	2.1	(1.2–3.9)	1.4	(1.0–2.2)	3.5	(2.3–8.6)	**0.001**
Serum sodium (mmol/L)	139	(135–143)	138	(135–142)	140	(135–145)	0.181
Serum potassium (mmol/L)	4.2	(3.7–4.7)	4.1	(3.6–4.6)	4.2	(3.8–4.8)	0.289
Hemoglobin (g/dL)	10.8	(9.0–12.5)	10.7	(9.0–12.9)	10.8	(9.0–12.3)	0.691
WBC (10^6^/mL)	13.13	(8.45–17.76)	13.06	(8.21–17.58)	13.97	(8.92–19.62)	0.657
Platelets (10^6^/mL)	201	(132–281)	212	(136–280)	197	(119–297)	0.649
INR	1.18	(1.08–1.32)	1.13	(1.06–1.23)	1.28	(1.12–1.64)	**0.001**
Fibrinogen (g/L)	4.40	(2.80–5.86)	4.93	(3.40–6.34)	3.67	(2.53–5.61)	0.074
D-dimer (µg/L)	4.13	(1.50–15.25)	2.68	(1.28–10.34)	11.59	(4.04–30.54)	**0.001**
AST (U/L)	61	(35–147)	50	(29–83)	85	(46–247)	**0.003**
ALT (U/L)	33	(18–97)	30	(18–87)	39	(17–125)	0.352
Bilirubin (mg/dL)	0.85	(0.50–1.36)	0.74	(0.49–1.31)	0.97	(0.53–1.54)	0.224
Troponin I (µg/L)	0.14	(0.03–0.92)	0.08	(0.02–0.37)	0.37	(0.05–1.73)	**0.002**
NT-pro BNP (pg/mL)	2794	(913–7978)	2256	(668–7053)	4500	(1033–12,742)	0.085
Procalcitonin (ng/mL)	2.44	(0.57–17.85)	1.65	(0.50–9.78)	5.66	(0.74–26.68)	**0.042**
CRP (mg/L)	144	(76–225)	147	(87–226)	137	(47–221)	0.204
**Primary endpoint**							
All-cause mortality at 30 days, *n* (%)	80	(49.4)	39	(41.9)	41	(59.4)	**0.028**
**Follow up data**, *n* (%)							
ICU time (days; median; (IQR))	7	(3–17)	8	(3–18)	5	(3–16)	0.098
Death ICU, *n* (%)	75	(46.3)	33	(35.5)	42	(60.9)	**0.001**

ALT, alanine aminotransferase; APACHE II, acute physiology and chronic health evaluation II; AST, aspartate aminotransferase; CRP, C-reactive Protein; DIC, disseminated intravascular coagulation; GFR, glomerular filtration rate; ICU, intensive-care unit; INR, international normalized ratio; IQR, interquartile range; NT-pro BNP, aminoterminal pro-B-type natriuretic peptide; SARS-CoV-2, severe acute respiratory syndrome coronavirus type 2; SOFA, sepsis-related organ failure assessment score; WBC, white blood cells. Level of significance between sepsis and septic shock *p* < 0.05. Bold type indicates statistical significance.

**Table 3:** Univariate correlations of TNI and BNP with laboratory and clinical parameters in all patients (*n* = 162) on day 1.

	cTNI		NT-Pro BNP	
	r	*p*-Value	r	*p*-Value
Age	0.092	0.293	0.222	**0.018**
BMI	0.000	0.997	−0.137	0.155
Hb (g/dL)	−0.045	0.607	−0.080	0.399
WBC (10^6^/mL)	−0.050	0.571	−0.159	0.091
Platelets (10^6^/mL)	−0.160	0.067	−0.225	**0.016**
Albumin (g/L)	−0.064	0.520	−0.170	0.112
Bilirubin (mg/dL)	0.072	0.493	0.185	0.088
cTNI (µg/L)	-	-	0.528	**0.001**
NT-pro BNP (pg/mL)	0.528	**0.001**	-	-
LVEF	0.307	**0.001**	0.439	**0.001**
CRP (mg/L)	−0.141	0.110	0.081	0.403
PCT (ng/mL)	0.209	**0.029**	0.247	**0.012**
PaO_2_/FiO_2_ ratio	0.115	0.201	0.202	**0.037**
Mechanical ventilation days	−0.053	0.544	−0.342	**0.001**
Creatinine (mg/dL)	0.154	0.078	0.291	**0.002**
Renal replacement days	0.053	0.545	−0.021	0.824
SOFA score	0.201	**0.020**	0.226	**0.015**
Acute Physiology score	0.239	**0.006**	0.192	**0.040**
APACHE II score	0.220	**0.011**	0.281	**0.002**
MAP (mmHg)	0.013	0.884	−0.199	**0.034**
Catecholamine use	0.133	0.127	−0.074	0.432
Intensive-care days	−0.080	0.358	−0.304	**0.001**

APACHE II, acute physiology and chronic health evaluation II; BMI, body mass index; CRP, C-reactive protein; cTNI, cardiac troponin I; Hb, hemoglobin; LVEF, left ventricular ejection fraction; MAP, mean arterial pressure; NT-pro BNP, aminoterminal pro-B-type natriuretic peptide; PCT, procalcitonin; SOFA, sepsis-related organ failure assessment; WBC, white blood cell count. Level of significance *p* < 0.05. Bold type indicates statistical significance.

**Table 4:** C-statistic for biomarkers at days 1, 2 and 3 to discriminate between patients with sepsis and septic shock.

	cTNI	NT-Pro BNP	*p*-Value for AUC Difference
**Day 1**	0.658 (0.564–0.753); ***p* = 0.002**	0.595 (0.488–0.702); *p* = 0.085	0.389
Day 1: Controls *n* = 93 patients with sepsis			
**Day 2**	0.547 (0.382–0.712); *p* = 0.592	0.517 (0.284–0.750); *p* = 0.888	0.842
Day 2: Controls *n* = 103 patients with sepsis			
**Day 3**	0.885 (0.770–1.000); ***p* = 0.001**	0.481 (0.162–0.800); *p* = 0.903	**0.022**
Day 3: Controls *n* = 100 patients with sepsis			

Level of significance *p* < 0.05. Bold type indicates statistical significance.

**Table 5:** C-statistic for biomarkers at days 1, 2 and 3 to discriminate between non-survivors and survivors.

	cTNI	NT-Pro BNP	*p*-Value for AUC Difference
**Day 1**	0.635 (0.541–0.729); ***p* = 0.007**	0.582 (0.477–0.687); *p* = 0.132	0.462
**Day 2**	0.687 (0.540–0.834); ***p* = 0.021**	0.537 (0.317–0.757); *p* = 0.735	0.255
**Day 3**	0.633 (0.436–0.830); *p* = 0.200	0.525 (0.315–0.735); *p* = 0.813	0.455

Level of significance *p* < 0.05. Bold type indicates statistical significance.

**Table 6:** Uni- and multivariable Cox regression analyses within the entire study cohort.

Variables	Univariable			Multivariable		
	HR	95% CI	*p*-Value	HR	95% CI	*p*-Value
Age	1.013	0.995–1.030	0.151	0.994	0.966–1.023	0.688
Sodium (mmol/L)	1.027	0.998–1.057	0.066	1.023	0.950–1.102	0.548
Potassium (mmol/L)	0.989	0.736–1.329	0.944	0.669	0.354–1.266	0.217
pH	0.101	0.015–0.686	**0.019**	0.067	0.002–2.793	0.155
WBC (10^6^/mL)	0.978	0.954–1.002	0.069	0.982	0.940–1.026	0.424
Platelets (10^6^/mL)	0.998	0.996–1.000	**0.042**	0.998	0.995–1.001	0.179
Malignancy	1.255	0.786–2.005	0.341	3.439	1.389–8.511	**0.008**
Immunosuppression	0.559	0.243–1.287	0.172	0.520	0.106–2.540	0.419
Respiratory rate > 22/min	0.793	0.511–1.231	0.301	0.696	0.306–1.582	0.387
Heart rate > 100 bpm	0.923	0.595–1.431	0.721	0.549	0.236–1.276	0.163
Systolic BP < 100 mmHg	0.851	0.520–1.392	0.520	1.457	0.619–3.429	0.389
Creatinine (mg/dL)	1.004	0.896–1.126	0.940	1.035	0.847–1.266	0.735
LVEF < 35%	1.239	0.747–2.055	0.407	4.084	1.475–11.304	**0.007**
cTNI > 0.136 µg/L	1.703	1.030–2.814	**0.038**	2.251	1.017–4.981	**0.045**
NT-pro BNP > 2793.5 pg/mL	1.769	1.048–2.986	**0.033**	1.364	0.528–3.521	0.522

cTNI, cardiac Troponin I; GCS, Glasgow coma scale; LVEF, left ventricular ejection fraction; NT-pro BNP, aminoterminal pro-B-type natriuretic peptide; Systolic BP, systolic blood pressure; WBC, white blood cell count. Level of significance *p* < 0.05. Bold type indicates statistical significance.

## Data Availability

The datasets used and/or analyzed during the current study are available from the corresponding author upon reasonable request.
